# Seeking allergy when it hides: which are the best fitting tests?

**DOI:** 10.1186/1939-4551-6-11

**Published:** 2013-07-01

**Authors:** Cristoforo Incorvaia, Nicola Fuiano, Giorgio W Canonica

**Affiliations:** 1 Allergy/Pulmonary Rehabilitation, ICP Hospital, via Bignami, Milan, Italy; 2 Pediatric Allergy Service, ASL FG, via Ciaccia, Torremaggiore, Italy; 3 Allergy and Respiratory Diseases, DIMI, Department of Internal Medicine, University of Genoa, Largo R. Benzi, Genoa, Italy

**Keywords:** Rhinitis, Asthma, LgE tests, Nonallergic, Local allergic rhinitis, T-cell mediated hypersensitivity, Atopy patch test

## Abstract

In the common practice of respiratory allergy, the confirmation by IgE tests of the relationship between the occurrence and duration of symptoms and the exposure to specific inhalant allergens allows an aetiological diagnosis. However, to see patients with suggestive history but negative IgE tests is not rare, and this generally leads to a diagnosis of nonallergic rhinitis or asthma. In many cases, such diagnosis is wrong, because the patient may be revealed as allergic by using additional testing. This is true for local allergic rhinitis, characterized by an exclusive IgE production in the nasal mucosa, that may be correctly diagnosed by performing a nasal IgE measurement or a nasal provocation test with the suspected allergen (s). Another misleading issue is the role of T cell-mediated, delayed hypersensitivity in the pathophysiology of rhinitis and asthma. Recent studies showed that in patients with rhinitis or asthma and negative IgE tests, especially when there is a positive history for current or past atopic dermatitis, the clinical symptoms are actually driven by such mechanism, that may be detected by performing an atopy patch test (APT). The allergen source most frequently responsible for this kind of allergy is the house dust mite, but other allergens may also be involved. Thus, before delivering a diagnosis of nonallergic rhinitis or asthma in patients with negative result to common allergy testing, further tests are needed. To miss the diagnosis of allergy has obvious consequences in terms of management, including allergen avoidance, patient’s education, and specific immunotherapy.

## Introduction

The diagnosis of allergy can be easy, as in the case of a patient with symptoms of rhinoconjunctivitis in the months of May and June and positive results to skin prick tests (SPT) with grass pollen extracts. However, two main issues make such an optimal combination relatively rare. The first is the natural history of respiratory allergy, which shows how a first sensitization, often occurring in childhood [1], is commonly followed by subsequent sensitizations to other allergens which define the polysensitization profile of the average adult patient [2-4]. To further confound this issue, sensitizations, as assessed by SPT or in vitro tests, may or may not be associated to clinical allergy. The simple sensitization not accompanied by clinical symptoms is defined as asymptomatic sensitization [5], but to distinguish the kind of sensitization in a patient with prolonged duration of symptoms and multiple positivities to allergy tests may reveal difficult. The modern approach of the component resolved diagnosis, based on testing the purified single allergen molecules, and requiring the knowledge of their characteristics as primary genuine sensitizers or simply cross-reacting components with no clinical significance, can remarkably help in identifying the true causative allergens [6,7].

The second issue stands on the fact that the common allergy tests are unable to detect all cases of allergy for two main reasons: (i) IgE antibodies may be present exclusively in the mucosal tissue, as occur in local production of IgE [8], or (ii) the allergic symptoms are sustained by non-IgE mediated mechanisms. To miss testing such possibility means to erroneously classify the patient as nonallergic and consequently to manage improperly his/her disease.

### Testing for local allergic rhinitis

The first recognitions that IgE may be present exclusively in the nasal mucosa date back to the 1970s [9,10] and this phenomenon was occasionally reconsidered [11-14]. In 2003 the term “entopy” was proposed to differentiate local IgE production from atopy [13] but the recent definition by Rondon et al. as local allergic rhinitis (LAR) seems more convincing [15]. The same authors evaluated the prevalence of LAR by a survey on 3860 patients attending their allergy service in one year, 452 of whom were selected for rhinitis [16]. An overall number of 428 patients completed the study; 24 were excluded because of nasal hyperreactivity. LAR was diagnosed in 25.7%, allergic rhinitis (AR) in 63.1%, and nonallergic rhinitis (NAR) in 11.2%. LAR was diagnosed on the basis of negative SPT and serum sIgE and positive nasal provocation test (NPT). In more than one third of LAR patients the onset of rhinitis was in childhood. The house dust mite (HDM) was the main sensitizing aeroallergen both in LAR and AR (60% and 54%, respectively). This finding highlights that LAR is not rare among patients with rhinitis, therefore to overlook its search would lead to the non-diagnosis of allergy in a substantial number of patients undergoing allergologic evaluation. One method to diagnose LAR is, as proposed by Rondon, the NPT, but a technique detecting IgE in nasal mucosa, introduced by Marcucci and Sensi [12] is also available. With such nasal IgE test, the allergen coupled cellulose derivative is placed in a two-hole applicator strip covered with a permeable membrane (to avoid the adhesion of nasal mucus on the substrate) and positioned in the above-posterior tract of the internal ostium for 10 minutes. The results are subsequently read according to a colorimetric reaction and expressed in a scale from 0 (negative) to 4 (highly positive), according to a calibration curve. A recent study compared the results of NPT and nasal IgE test in 55 children with rhinitis in the periods when Alternaria spores are present in the air. A concomitant positivity of NPT and nasal IgE test to Alternaria was observed in about 70% of patients, while positivity of SPT and NPT was observed in 27% of patients, this difference being highly significant (p < 0.0001). This suggests that sensitisation to Alternaria is frequently expressed by exclusive production of specific IgE in the nasal mucosa [17]. Of note, a clinically relevant polysensitization to aeroallergens may occur also in patients with LAR and may be detected by multiple NPT [18].

The pathophysiology of LAR is not fully elucidated, however a number of possible mechanisms underlying the local production of IgE were reported. Among them, it was found that nasal B cells express epsilon-germline gene transcripts and mRNA for the epsilon heavy chain of IgE [19]. In patients with negative result to IgE tests a Th2 inflammatory pattern, with increased number of IgE + B cells, mast cells and eosinophils, was detected by *in situ* hybridization [20]. A nasal Th2 IgE-mediated inflammation was specifically confirmed in patients with LAR, in whom cytometric evaluation in nasal lavage showed a leukocyte-lymphocyte phenotype with increased number of mast cells, basophils, eosinophils, CD3+ T cells and CD4+ T cells during natural exposure to the causative allergen (grass pollen) comparable to that of patients with AR [21]; in the same study, the NPT with grass pollen elicited an immediate response (associated to release of tryptase) and a late response, as occurs in AR [21]. This pattern was confirmed also in patients with mite-induced perennial LAR [22]; in both studies, the rapid and persistent rise of specific IgE from the baseline following NPT supports the local origin of the IgE production. What remains to be determined are the factors differentiating the systemic and local IgE synthesis occurring in the common patient with AR from the exclusive local synthesis occurring in the patient with LAR. Of note, it was recently reported that in patients with positive IgE tests but with no clinical symptoms, i.e. the patient with asymptomatic atopy, there are no local IgE in the nasal mucosa, and it seems conceivable that this should account for being asymptomatic. In fact, patients with local but not systemic IgE, that is, with negative results to IgE tests, have clinical symptoms, while patients with systemic but not local IgE are asymptomatic [23].

### Testing for a cell-mediated mechanism

Recent research has provided evidence that T-cell mediated reactions to inhalant allergens may sustain respiratory symptoms in patients with negative results to SPT and in vitro IgE tests. The major cause of this kind of reaction is the HDM, particularly *Dermatophagoides pteronyssinus* and *Dermatophagoides farinae* (belonging to the family *Pyroglyphidae*) which produce a wide array of allergens occurring in mite bodies and faeces. Currently, there are 22 defined mite allergens, some of them acting as major allergens based on the recognition by IgE from more than 50% of mite allergic patients. Most of these allergens are proteolytic enzymes, as known for Der p 1 and Der p 2 from *D. Pteronyssinus* and Der f 1 and Der f 2 from *D. farinae*[24]. The proteolytic activity makes *Dermatophagoides* particularly efficient in sensitizing through the skin. In particular, subjects (and especially children) with atopic dermatitis (AD) have a baseline-impaired barrier function that allows proteins to enter into the viable epidermis [25] and to work as enhancers of the barrier impairment and triggers of a IgE response. In the epidermal barrier dysfunction occurring in AD a pivotal role is played by filaggrin [26]. Filaggrin, which derives from the highly phosphorylated polypeptide profilaggrin, the main constituent of the keratohyalin substance in the granular layer, is a structural protein associated to filaments which are bound to keratin fibres in epidermal cells. Recent studies found that loss-of-function mutations in the gene encoding filaggrin, particularly the R501X and 2282de14 mutation, are associated with the development of AD [27,28]. In this condition, the airborne proteins produced by HDM, such as cysteine and serine proteases, have direct proteolytic activity on the skin that contribute to barrier impairment and delayed barrier recovery in patients with AD [29], by disrupting epithelial tight junctions, by inducing degranulation of eosinophils, and by activating keratinocytes, that leads to increased production of interleukin (IL)-6, IL-8, and granulocyte-macrophage colony-stimulating factor (GM-CSF) [30,31]. The altered barrier function facilitates the interaction between allergens and the local immune cells to initiate the type-I-immediate and type-IV-delayed hypersensitivity reactions common among patients with AD [32]. These mechanisms account for the major role currently recognized to HDM in AD [33]. However, in AD the reactivity to HDM allergens is more often cell-mediated than IgE-mediated, and this limits the diagnostic role of SPT and in vitro IgE tests. Instead, the atopy patch test (APT), introduced in 1989 by Ring et al. as a tool to investigate the role of aeroallergens in AD [34], properly assesses the type-IV delayed hypersensitivity, based on notable evidence supporting its capacity to reproduce the pathophysiologic events of AD. In biopsy-based studies, a Th2 cytokine pattern was found 24 hours after APT, but a shift to a Th1 pattern, as occurs in chronic AD skin lesions, was noted after 48 hours [35,36]. A more frequent positivity to APT was reported in patients with allergen-specific lymphocyte proliferation and expression of activation markers on peripheral blood T-cells following in vitro stimulation with HDM, but also with cat or grass pollen allergens, than in patients without lymphocyte proliferation [37]. Application of the APT to skin of subjects with AD was followed by an influx of inflammatory dendritic epidermal cells [38]. By immunohistochemical analysis, the presence of IgE on Langerhans cells was demonstrated in positive APT reactions to HDM in patients with mite-associated AD [39]. Clinically, patients with a diagnosis of intrinsic AD because of negative IgE tests actually had a positive APT for dust mites [40]. This aspect is of particular interest, because AD patients with negative SPT and IgE measurement in serum should be classified as nonatopic unless APT is performed. In a European multicenter study, which included 314 patients with AD, the frequency of positive APT reactions to HDM was 39%, and a positive APT without SPT or sIgE for the respective allergen was seen in 17% of the patients [41]. New significant observations increased the value of the APT by showing that in children with respiratory symptoms an exclusive positivity to APT with HDM can be detected [42]. This is also true in children with mite-induced asthma and rhinitis with positive SPT and specific IgE in serum, in 25% of cases a positive APT to HDM is found, indicating that delayed hypersensitivity was concurrent to immediate hypersensitivity [43]. Further studies investigated the possible factors underlying the positive result of APT in subjects with respiratory symptoms. In a study conducted on 297 children it was demonstrated that in subjects with asthma or rhinitis a positive APT to HDM was strongly associated with the presence of current or past AD, while most subjects with respiratory disease but a negative history for AD had a positive SPT [44]. Multivariate analysis showed a highly increased likelihood of a positive APT result in patients with AD (odds ratio 17.4), in patients with AD and respiratory disease (odds ratio 21.9), and in patients with past AD and respiratory disease (odds ratio 22.8). These observations were confirmed in a study on a large population of 465 children, divided into 4 groups: group A, current AD (40 patients); group B, current AD with respiratory symptoms (156 patients); group C, past AD with respiratory symptoms (203 patients); and the control group, respiratory symptoms with no history of AD (66 patients). The APT was significantly more frequently positive in groups with current AD (groups A and B) or past AD (group C) than in the control group, while SPT and specific IgE in serum were significantly more frequently positive in the control group [45]. Such significant differences in response to APT in patients with dissimilar clinical expressions suggest that distinctive immunologic mechanisms underlie the different manifestations of hypersensitivity to dust mites. One may conceive that in subjects with a negative history for AD sensitization to mites occurs by respiratory route and leads to the development of a Th2 pattern of response with ongoing production of specific IgE and consequent positive SPT and in vitro IgE tests. By contrast, if the mite allergens enter through the skin, as can occur during exposure to common indoor concentrations of the major allergen Der p 1 [46], such entering being facilitated by its proteolytic activity and in the presence of a skin barrier dysfunction, a different chain of events is likely to take place, which is revealed by positive APT and negative SPT and in vitro IgE tests. This knowledge should change the attitude of allergy specialists to using the ATP, especially when patients with rhinitis or asthma, positive history for past AD, and negative IgE test are to be evaluated [47]. However, thus far there are no studies demonstrating that in patients with negative IgE tests there is an isolated late response to allergen challenge. Further studies should assess the diagnostic value of the APT by comparing its result with the effect of allergen exposure (better if proved by a nasal challenge). A correlation between the results of the two tests should confirm the reliability of the APT.

### How to manage patients with suspected allergy and negative IgE tests

**Figure 1:**
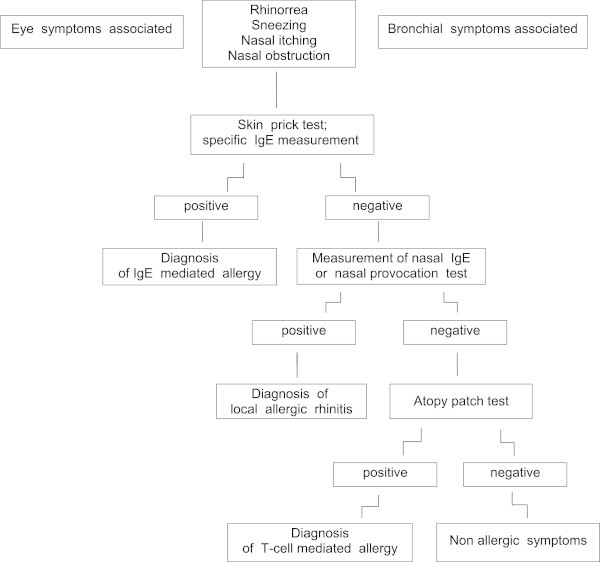
Diagnostic flow-chart to detect forms of allergy different from the common IgE-mediated hypersensitivity.

It is common knowledge that not all patients with rhinitis or asthma are allergic. Concerning the latter, “intrinsic” asthma is estimated in 10 to 33% of all patients with asthma [48], while nonallergic rhinitis seems to affect approximately 8% of the general population in the USA [49], this figure being close to the 11% in Spain as reported by Rondon et al. in the study cited above [16]. In both diseases, it can be argued that if the allergologic evaluation is not limited to SPT and in vitro IgE tests, the number of patients diagnosed as nonallergic could be substantially reduced. To diagnose LAR, which currently is a well defined clinical entity, a NPT or a measurement of nasal IgE are required. For the latter, a recent study showed that using molecular diagnosis, with identification of IgE to single allergen components, may strengthen the significance of the findings [50]. In addition, in recent years the possibility that rhinitis or asthma are sustained by a T-cell mediated mechanism has gained increasing recognition. The picture suggesting such mechanism is characterized by a positive history for past or current AD and negative IgE tests. The test required to establish that a T-cell mediated, delayed hypersensitivity, is driving the clinical symptoms of the patient, is the APT. The allergen source most frequently responsible for T-cell mediated rhinitis or asthma is the HDM, but other allergens may also be involved [47]. Thus, in patients with positive history and negative skin tests or in vitro IgE tests, testing for LAR or T-cell mediated allergy should be added as third level examination, using a flow-chart as suggested in Figure 1. To reach an accurate diagnosis of allergy by using the correct tests in a patient otherwise classified as nonallergic has obvious consequences in terms of management, including allergen avoidance, patient’s education, and specific immunotherapy. For example, a preliminary study on 20 patients with grass pollen-induced seasonal LAR, 10 treated with subcutaneous immunotherapy with a grass-pollen extract and 10 with symptomatic drugs, reported that immunotherapy was effective, as assessed by their symptoms score during the pollen season, and well tolerated; in addition, immunologic effects on IgG and IgE antibodies, comparable to the pattern of response observed in patients with AR, were found [51]. A confirmation of such outcome from controlled trials including an adequate number of patients should make more convincing this issue.

The feasibility of immunotherapy deserves to be investigated also in patients with rhinitis or asthma and evidence of a T-cell mediated hypersensitivity as indicated by positive APT.

## Competing interests

The authors declare that they have no competing interests.

## Authors’ contributions

CI, NF and GWC equally contributed to writing the manuscript. All authors read and approved the final manuscript.
